# The smell of love in Drosophila

**DOI:** 10.3389/fphys.2013.00072

**Published:** 2013-04-05

**Authors:** Anna B. Ziegler, Martine Berthelot-Grosjean, Yael Grosjean

**Affiliations:** ^1^CNRS, Centre des Sciences du Goût et de l'Alimentation, UMR-6265Dijon, France; ^2^INRA, Centre des Sciences du Goût et de l'Alimentation, UMR-1324Dijon, France; ^3^UMR, Centre des Sciences du Goût et de l'Alimentation, Université de BourgogneDijon, France

**Keywords:** courtship, Drosophila, olfaction, receptor, nervous system

## Abstract

Odors are key sensory signals for social communication and food search in animals including insects. *Drosophila melanogaster*, is a powerful neurogenetic model commonly used to reveal molecular and cellular mechanisms involved in odorant detection. Males use olfaction together with other sensory modalities to find their mates. Here, we review known olfactory signals, their related olfactory receptors, and the corresponding neuronal architecture impacting courtship. OR67d receptor detects 11-cis-Vaccenyl Acetate (cVA), a male specific pheromone transferred to the female during copulation. Transferred cVA is able to reduce female attractiveness for other males after mating, and is also suspected to decrease male-male courtship. cVA can also serve as an aggregation signal, maybe through another OR. OR47b was shown to be activated by fly odors, and to enhance courtship depending on taste pheromones. IR84a detects phenylacetic acid (PAA) and phenylacetaldehyde (PA). These two odors are not pheromones produced by flies, but are present in various fly food sources. PAA enhances male courtship, acting as a food aphrodisiac. Drosophila males have thus developed complementary olfactory strategies to help them to select their mates.

Finding a sexual partner is the primary quest necessary for reproduction in most animal species. Over the past 60 years, and thanks to the pioneer works of researchers like Herman T. Spieth, Jeffrey C. Hall, and others, *Drosophila melanogaster* has emerged as a powerful model to tackle the neurogenetic basis of reproductive behaviors such as courtship (Spieth, 1952; Hall, 1977; Villella and Hall, 2008). Courtship in Drosophila is a relatively stereotyped ritual, easy to observe especially in regard to male actions. Classically, the male orients toward the object and if it looks promising (for example, if it is a virgin female), he will tap her abdomen with his front legs to detect specific cuticular pheromones. The male will also vibrate one of his wings to produce a courtship-specific sound to seduce his mate. He will lick her genitalia to taste for chemicals, and finally will try to copulate (Spieth, 1952, 1966, 1974; Greenspan and Ferveur, 2000). Recently, it has been shown that quivering of the male abdomen coincides with female immobility and therefore with her receptivity, much more than male wing fluttering does (Fabre et al., 2012). Females were classically described as a rather passive partner during male courtship, although they perform subtle rejection-like behaviors (Connolly and Cook, 1973). The female contribution to courtship sequence is still poorly studied and understood (Ferveur, 2010). But only if she agrees, she will facilitate the copulation by slowing down her locomotor activity and opening her genitalia (Hall, 1994). Detailed analyses of the courtship ritual between partners showed that this sequence of stereotyped behaviors is not linear but rather very complex, and somehow reflect a sophisticated dialog between partners (Lasbleiz et al., 2006). Finally, once a female has copulated, physiological and behavioral changes occur within her (Wolfner, 1997, 2002), which will change her reactions to courtship and her attractiveness for males (Mehren et al., 2004; Rezaval et al., 2012).

*Drosophila melanogaster* flies use a wide range of sensory modalities to discriminate between their potential mates. These include vision to track the partner, hearing to detect mate song, and chemoperception of pheromones through taste and olfaction (Greenspan and Ferveur, 2000). These sensory signals are detected through peripheral sensory “organs” (proboscis, leg tarsae, wings, eyes, antennae, maxillary palps; Figure 1A). These appendages house sensory neurons. A proportion of these sensory neurons are similar between both sexes. Some others have gender-specific characteristics. Two genes encoding transcription factors are known to be crucial for cell gender identity leading to sex-specific behaviors: *fruitless* (*fru*; Gailey and Hall, 1989; Ito et al., 1996; Ryner et al., 1996) and *doublesex* (*dsx*; Rezaval et al., 2012). Both of them regulate the formation of a sexually dimorphic brain. *dsx* is not known to play a role in olfactory system development or function, but it acts in gustatory sensory organ precursors specification, and in GR68a taste receptor expression (Bray and Amrein, 2003; Mellert et al., 2012). *fru* is the most studied for its impact on courtship. This gene produces several transcripts coding for transcription factors, and one of them is male specific: Fru^M^. Fru^M^, which is only expressed in a specific subset of neurons, is supposed to be a master factor leading to male specific behaviors (Manoli et al., 2005; Stockinger et al., 2005), even if its exact role is controversial (Villella and Hall, 2008). Here, we review known olfactory signals and the related olfactory neuronal architecture impacting courtship in males.

**Figure 1:**
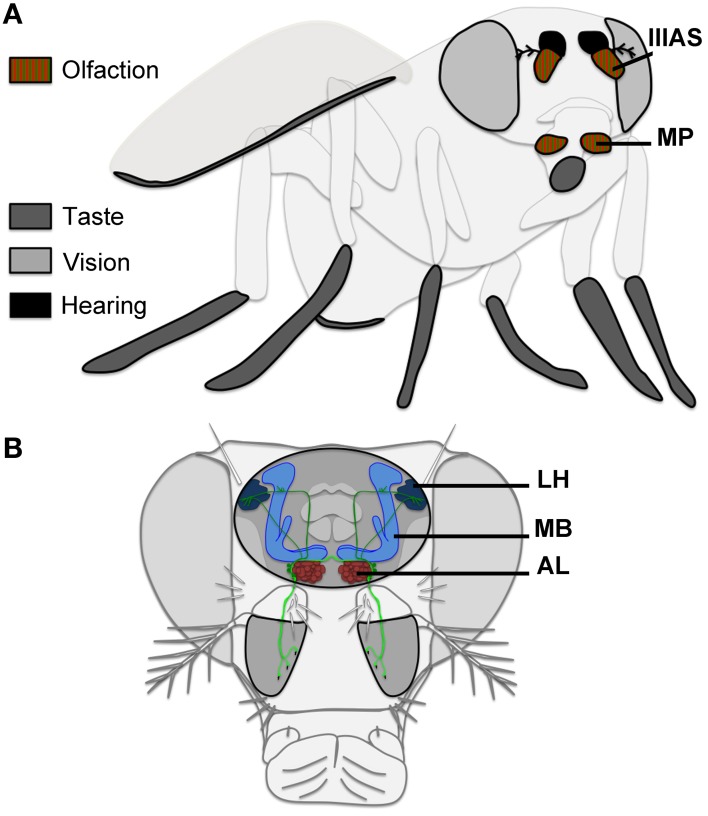
**Olfactory perception in Drosophila. (A)** Olfactory signals are detected through the maxillary palps (MP) and the 3rd antennal segment (IIIAS). **(B)** OSNs (light green) project to the antennal lobe (AL), where the signal is transferred to projection neurons (dark green) targeting the mushroom bodies (MB) and to the lateral horn (LH).

## Olfactory perception in drosophila

Drosophila olfactory perception is based on two main classically described paired-appendages located on the drosophila head: the 3rd-antennal segments (also called funiculi, including the sacculi, pits located on the posterior side of these appendages), and the maxillary palps (Figure 1; Nayak and Singh, 1985; Stocker, 1994; de Bruyne et al., 1999; Hallem and Carlson, 2006; Ai et al., 2010). The funiculus houses 3 types of olfactory sensilla: basiconic, trichoid, and coeloconic sensilla, whereas the maxillary palp has only basiconic sensilla (Stocker, 1994). The arista, which is the distal part of the antenna, could also be involved in olfactory perception (Thorne and Amrein, 2008; Benton et al., 2009), although it was suggested to play a role in thermoperception (Stocker, 1994). This arista has also a role in the detection of acoustic vibrations from neurons whose cell bodies are located in the 2nd segment of the antenna (Johnston's organ; Kamikouchi et al., 2009). The possible role of the arista in olfactory perception is still largely unknown.

Olfactory sensory neurons (OSNs) are located within the sensilla (usually two to three neurons per sensilla, depending on the sensilla type). Each type of OSN expresses a specific class of olfactory receptors: either ORs (seven transmembrane domain receptors, associated with basiconic, and trichoid sensilla), or IRs (ionotropic receptors related to ionotropic glutamate receptor family, associated with coeloconic sensilla). These two families of olfactory receptors are in fact complexes, where a specific OR or IR is associated with a cofactor: either ORco (also called OR83b), or IR25a or IR8a, to form a functional ligand-gated ion channel (Vosshall et al., 1999; Couto et al., 2005; Hallem and Carlson, 2006; Sato et al., 2008; Benton et al., 2009; Abuin et al., 2011; Silbering et al., 2011). Some exceptions exist to this general rule. For example, some olfactory neurons detecting CO_2_ express GR21a and GR63a, which belong to the family of gustatory receptors (Suh et al., 2004; Jones et al., 2007).

**Figure 2:**
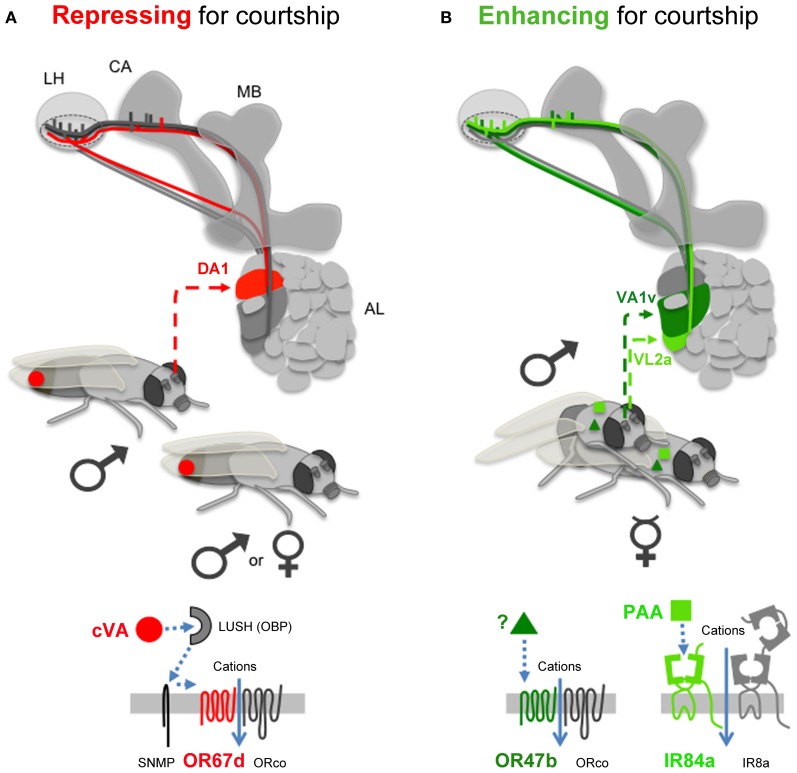
**Olfactory pathways influencing courtship. (A)** The only known repressing olfactory signal comes from cVA (red circle, present into male, and into mated female sex-organs; Farine et al., 2012), which is detected by OR67d. LUSH is an odorant binding protein (OBP), which interacts with Sensory neuron membrane protein (SNMP), to activate OR67d. OSNs expressing OR67d project to the DA1 glomerulus in the AL. The olfactory information is then transferred to projection neurons targeting the MB (in the calyx region, CA), and the LH. **(B)** Enhancing signals (present on the flies such as virgin females) stimulate either OR47b (with an unknown odorant, dark green triangle), or IR84a (with PAA, light green square). OR47b OSNs target the VA1v glomerulus, and IR84a OSNs VL2a. Projection neurons emerging from these three AL glomeruli project into a specific area of the LH (doted circle). Symbols: ♂ for male, ♀ for mated female, 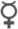 for virgin female.

Only three different types of OSNs in males express Fru^M^, making them the best candidates to detect fly volatile pheromones potentially impacting courtship. These OSNs project to three different glomeruli within the antennal lobe (primary olfactory center): OSNs expressing OR67d target the DA1 glomerulus; OR47b OSNs target the VA1v glomerulus; and IR84a OSNs target the VL2a glomerulus (Stockinger et al., 2005; Grosjean et al., 2011; Figures 1B, 2). These three glomeruli are also significantly larger in males than in females, suggesting a sex-specific role for each of them in olfactory behavior (Kondoh et al., 2003; Stockinger et al., 2005). Projection neurons (PNs) emerging from these three glomeruli then reach the mushroom bodies, and the lateral horn (secondary olfactory centers; Jefferis et al., 2007; Grosjean et al., 2011; Figure 2). Interestingly, the DA1 projection neurons project into the lateral horn in a sexually dimorphic manner (Datta et al., 2008; Ruta et al., 2010). The mushroom bodies represent a key center for olfactory memory also important for male courtship (Neckameyer, 1998; Heimbeck et al., 2001; Heisenberg, 2003; Sakai et al., 2012), whereas the lateral horn is supposed to mediate innate behaviors (Jefferis et al., 2007). PNs coming from DA1, VA1v, and VL2a are all targeting a segregated region within the lateral horn, potentially involved in pheromonal signal processing. VA1v and VL2a are slightly more clustered together than PNs coming from DA1 (Jefferis et al., 2007; Grosjean et al., 2011; Figure 2).

## cVA, an olfactory signal inhibiting male courtship, but not only

11-cis-Vaccenyl Acetate (cVA) is the only volatile inhibitory pheromone for male courtship in *Drosophila melanogaster* identified yet (Jallon et al., 1981). cVA is synthesized by males. The male transfers this lipid to the virgin female during copulation through his genital organ (Butterworth, 1969; Brieger and Butterworth, 1970). One olfactory receptor, OR67d, has been shown to detect cVA. cVA regulates both male and female mating behavior through OR67d (Kurtovic et al., 2007). When males sense cVA from other males or from mated females, they develop a generalized suppression of courtship (Ejima et al., 2007). Interestingly cVA also promotes male-male aggression (Wang and Anderson, 2010). In contrast, cVA/OR67d favors mating behavior in females (Figure 2A; Kurtovic et al., 2007).

The molecular sequence leading to the detection of cVA through OR67d to repress male courtship is now understood. cVA is a hydrophobic molecule, which needs an odorant binding protein (LUSH) to be soluble in the hemolymph, and to reach its receptor with the help of the SNMP co-factor (Figure 2A; Xu et al., 2005; Ha and Smith, 2006; Benton et al., 2007; Kurtovic et al., 2007; Jin et al., 2008; Laughlin et al., 2008).

OR65a is also known to be able to detect cVA (van der Goes van Naters and Carlson, 2007). Recently, Liu and collaborators have shown that OR65a artificial activation is sufficient to reduce aggression between males (Liu et al., 2011). It has also been proposed that OR65a could be involved in cVA's inhibitory role on male courtship, instead of OR67d (Ejima et al., 2007). This second possible role of OR65a has to be clarified since it does not represent the currently favored pathway.

cVA can also act as an attracting long-range volatile signal when associated with food odors (Bartelt et al., 1985). We do not know yet which olfactory molecular and neuronal pathway is responsible for this behavior.

## Olfactory signals enhancing male courtship

IR84a together with IR8a cofactor forms a functional ionotropic channel (Abuin et al., 2011), which detects phenylacetic acid (PAA) and phenylacetaldehyde (PA; Benton et al., 2009; Grosjean et al., 2011; Silbering et al., 2011). These two volatile chemicals are not pheromones produced by flies, but rather are present in various fly food sources (Grosjean et al., 2011). Both aromatic compounds are present on fruit and plants (Wightman and Lighty, 1982), as well as in their fermentation products (Barata et al., 2011). PAA acts as a growth-regulating auxin in vegetal tissues (Wightman and Lighty, 1982), and is also synthesized by plant associated microorganisms such as yeast (Kim et al., 2007). Flies can perfume their body with PAA and PA when walking on the food (Grosjean et al., 2011).

PAA enhances male courtship through IR84a, acting as a food aphrodisiac (Figure 2B; Grosjean et al., 2011). Interestingly, PAA is not appealing on its own since it is not attractive using olfactory behavioral tests such as T- and Y-mazes (Grosjean et al., 2011; Silbering et al., 2011), but it has the potential to enhance male courtship toward other males or females (Grosjean et al., 2011). This suggests that the olfactory signal generated by PAA/IR84a is somehow working together with other sensory modalities (such as taste, hearing, or other olfactory cues) to stimulate male courtship.

The second olfactory receptor that enhances male courtship is OR47b. OR47b was shown to detect fly odors present on males and females (Figure 2B; van der Goes van Naters and Carlson, 2007). The exact identity of the OR47b ligand is still unknown. But the action of the OSNs expressing OR47b is dependent on the presence or absence of male-specific taste pheromones detected via taste neurons (through GR32a). Indeed, the increased courtship caused by depletion of male cuticular hydrocarbons (such as 7-tricosene) is suppressed by a mutation in OR47b, but the mutation of OR47b has no gross effect on male courtship when 7-tricosene is present (Wang et al., 2011). 7-tricosene is known to enhance male-male aggression, and is well described as an anti-aphrodisiac for male courtship (Savarit et al., 1999; Billeter et al., 2009; Wang et al., 2011). Thus, the influence of gustatory cues such as 7-Tricosene is dominant compared to olfactory signals detected by OR47b.

## Olfactory perception and courtship: what more could there be?

Drosophila males have developed complementary olfactory strategies to find their mates, based on inhibiting and/or stimulating olfactory signals. Enhancing olfactory cues for male courtship appears to be highly context-dependant (Grosjean et al., 2011; Wang et al., 2011). It would be very useful to know more about the integration process happening within male brains to compare sensory stimuli of different nature (olfactory, gustatory, auditory, visual).

Recently some cuticular hydrocarbons have been shown to be volatile (Farine et al., 2012). This suggests that they might also play a role through olfaction, and not exclusively via taste perception as previously believed. They could represent potential ligands for Fru^M^-positive OSNs, or for Fru^M^-negative ones. One possible receptor would be OR88a, since it detects fly odors (van der Goes van Naters and Carlson, 2007).

In summary, it appears increasingly clear that olfaction is not the main sensory modality for male courtship choice. Nevertheless, olfaction makes an important contribution to multimodal sensory inputs for the male choice to court or not to court.

### Conflict of interest statement

The authors declare that the research was conducted in the absence of any commercial or financial relationships that could be construed as a potential conflict of interest.
